# Effectiveness of peer support for improving glycaemic control in patients with type 2 diabetes: a meta-analysis of randomized controlled trials

**DOI:** 10.1186/s12889-015-1798-y

**Published:** 2015-05-06

**Authors:** Li Qi, Qin Liu, Xiaoling Qi, Na Wu, Wenge Tang, Hongyan Xiong

**Affiliations:** Department of Military Epidemiology, College of Military Prevention, Third Military Medical University, Chongqing, China; Chongqing Municipal Center for Disease Control and Prevention, Chongqing, China; School of Public Health and Management, Chongqing Medical University, Chongqing, China; Department of Dental Medicine, Sichuan University, Chengdu, China

**Keywords:** Peer support, Randomized control trial, Type 2 diabetes, Meta-analysis

## Abstract

**Background:**

To assess the effects of peer support at improving glycemic control in patients with type 2 diabetes.

**Methods:**

Relevant electronic databases were sought for this investigation up to Dec 2014. Randomized controlled trials involving patients with type 2 diabetes that evaluated the effect of peer support on glycated hemoglobin (HbA1c) concentrations were included. The pooled mean differences (MD) between intervention and control groups with 95% confidence interval (CI) were calculated using random-effects model. The Cochrane Collaboration’s tool was used to assess the risk of bias.

**Results:**

Thirteen randomized controlled trials met the inclusion criteria. Peer support resulted in a significant reduction in HbA1c (MD −0.57 [95% CI: −0.78 to −0.36]). Programs with moderate or high frequency of contact showed a significant reduction in HbA1c levels (MD −0.52 [95% CI: −0.60 to −0.44] and −0.75 [95% CI: −1.21 to −0.29], respectively), whereas programs with low frequency of contact showed no significant reduction (MD −0.32 [95% CI: −0.74 to 0.09]). The reduction in HbA1c were greater among patients with a baseline HbA1c ≥ 8.5% (MD −0.78 [95% CI: −1.06 to −0.51]) and between 7.5 ~ 8.5% (MD −0.76 [95% CI: −1.05 to −0.47]), than patients with HbA1c < 7.5% (MD −0.08 [95% CI: −0.32 to 0.16]).

**Conclusions:**

Peer support had a significant impact on HbA_1c_ levels among patients with type 2 diabetes. Priority should be given to programs with moderate or high frequency of contact for target patients with poor glycemic control rather than programs with low frequency of contact that target the overall population of patients.

**Electronic supplementary material:**

The online version of this article (doi:10.1186/s12889-015-1798-y) contains supplementary material, which is available to authorized users.

## Background

Diabetes (predominantly type 2 diabetes) ranks highly on the international health agenda as a global pandemic and as a threat to human health and global economies [1,2]. The self-management of diabetes, involving lifestyle modifications such as improving diet, increasing physical activity, self-monitoring of health status (blood glucose and examination of feet) as well as adherence to medication regimens, are key to improving outcomes in diabetes [3].

Specialist nurses and diabetes educators are being used to promote diabetes self-management [4-6], but such programs are resource intensive, the numbers of specialist nurses and diabetes educators are not adequate to carter for the increasing demand for diabetes care, especially in low-resource settings [7,8]. Therefore, it is urgent to find innovative and effective solutions that build on available resources to help patients successfully manage diabetes.

Currently, growing evidence suggests that peer support offers a promising solution. Peer support has been defined as ‘support from a person who possesses experiential knowledge of a specific behavior or stressor and similar characteristics as the target population [9]. Peer support helps reduce or prevent problematic health behaviours [10,11], vascular disease [12], HIV [13,14], Parkinson’s disease [15], etc. The success of peer support appears to be due in part to the nonhierarchical, reciprocal relationship that is created through the sharing of similar life experiences [9], and psychosocial processes that may be important in peer support including social support, experiential knowledge, and those described by social learning theory, social comparison theory and the helper–therapy principle [16]. Equally important, by training and employing non-professional staff members, peer support seems to be much less resource-intensive than traditional case management models. Therefore, it has been hypothesized that peer support could be considered as an alternative for diabetes self-management education and on-going support programs and tackling the burden of diabetes. To date, studies on the effect of peer support on patients with diabetes have shown inconsistent results [17,18].

Several reviews have been conducted, but these are focused solely on one type of peer support, such as that from a community health worker [19], volunteer-based peer support [20] or telephone peer support intervention [21], neither of which included randomized controlled trials (RCTs) [19-22] or quantitative analysis [23,24].

Consequently, we conducted a meta-analysis of RCTs to evaluate the effect of peer support on glycemic control among patients with type 2 diabetes (T2DM), which accounts for 90-95% of all diagnosed cases of diabetes. The results will facilitate the planning of evidence-based programs and will help inform future research.

## Methods

This meta-analysis is reported following the criteria of PRISMA statement [25] and was approved by ethical committee of research in Chongqing Municipal Center for Disease Control and Prevention.

### Data sources and searches

An extensive MEDLINE (from 1978 to Dec 2014), EMBASE (1980 to Dec 2014) and Cochrane Collaborative database (up to Dec 2014) were sought for RCTs based on the following search terms: peer support-related terms (‘peer’, ‘promoters’, ‘patient navigators’, ‘lay health workers’, ‘community health worker’, ‘peer educator’, ‘peer mentor’, ‘lay health leader’, ‘peer support’ and ‘natural helpers’) and diabetes-related terms (‘diabetes mellitus’, ‘T2DM’, ‘Glycosylated hemoglobin’, ‘HbA1c’, and ‘NIDDM’).

### Inclusion criteria and outcomes

Studies were considered eligible for the meta-analysis if they met the following inclusion criteria: 1) RCTs, because this study design has maximum validity and causal inference [26]; 2) adults (aged ≥18 years) diagnosed with T2DM; 3) studies that reported HbA1c levels, which is an index of the mean blood glucose concentration of the preceding 8–12 weeks and is the recommended index for evaluation of glycemic control of diabetes [27]; 4) peer support represented the majority of the interventions; 5) compared with a usual or routine care group.

Studies were excluded if they met one of the exclusion criteria: 1) the intervention was delivered by a health care professional; 2) the intervention did not involve direct contact between peer supporters and patient or was unclear; 3) non-English language publications.

### Methods of the review

Abstracts of cited articles were evaluated by two independent reviewers (QL & QXL) to determine the relevance, with differences resolved by a third reviewer (XHY) where necessary. When studies appeared to meet all the inclusion criteria, but data was incomplete, we contacted authors for additional data or clarification. Whenever there were multiple reports from the same trial, the most complete and/or more recently reported data were chosen.

### Data extraction

Two reviewers (QL & QXL) evaluated each study separately and extracted data. To assess the outcome, HbA1c levels before and after the intervention were noted. In the event of several post-intervention values, only the first one was considered. Other data extracted was as follows: characteristics of the participants (gender, age, HbA1c value at baseline and enrollment criteria), sample size, intervention mode (described in the following paragraph), frequency of contact, interval between pre- and post-intervention and the theory basis, etc.

Mode of peer support: In order to obtain relevant results for our meta-analysis, we divided peer supporters into two modes namely, ‘Community health workers’ and ‘Peer coaches’, based on literature reviews and expert opinions [16,22,24]. (i) Community health workers (CHWs) are members of the local community who serve as bridges between patients and health care providers [28], and they promote health in their communities through information distribution, assistance, social support and organization of community networks [29]. They have not necessarily had diabetes themselves but have been peers to the populations they serve in other important respects: They often speak the local language, share community, culture, and life experiences with their clients [29,30]. A number of different terms are used for CHWs including promoters, patient navigators, lay health workers and natural helpers. (ii) Peer coaches, also named as peer educators, peer mentors or peer leaders, are more informal and offer a flexible approach to provide peer support for patients. Peer coaches might be diabetes patients who have successfully coped with diabetes, and also could be those patients who had high HbA1c level and were struggling to bring down their glucose level [31].

In addition, frequency of contact was estimated on the basis of the reported intervention protocol and when available, the results. We classified the frequency into three levels: low (less than one contact in a period of one month per patient), moderate (one or two contacts in a period of one month per patient) and high (more than two contacts in a month per patient).

In the event of discrepancies in the data extracted, the same data was subjected to further review by another member of our team (XHY), and the consensus was arrived at.

### Assessment of the methodological quality of individual studies

Two members of our research team (QL & LQ) assessed each trial independently. We assessed risk of bias using the Cochrane Collaboration’s tool (Higgins [32]), regarding the following domains:

- Random sequence generation (selection bias);
- Allocation concealment (selection bias);
- Blinding (performance bias and detection bias) of outcome assessment. Because of the nature of the study design, it seems impossible to blind the participants, thus, blinding of participants was not be used as a criteria for risk of bias evaluation;
- Incomplete outcome data (attrition bias);
- Selective reporting (reporting bias);
- Other bias.

As recommended, we rated each item as: 1) “little risk of bias” if it is completely fulfilled quality standards with the least bias; 2) “unclear” if it is plausible that a bias raises some doubt about the results; and 3) “high risk of bias” if it is plausible that a bias seriously weakens confidence in the results. In cases of disagreement, the rest of the group was consulted, and judgment was made based on consensus. The assessment was not used as a criterion for the selection of trials, whereas some items were used only for descriptive purposes.

### Statistical analysis

Statistical analyses were conducted following the recommendations of the Cochrane Handbook for Systematic Reviews of Interventions and the PRISMA statement. We performed all analyses in Review Manager 5.2 and Stata version 12.1.

The meta-analysis was conducted using a random-effects model because of the a priori heterogeneity. The x^2^ and *I*^2^ statistics were used to assess statistical heterogeneity across studies, with *I*^2^ values of 50% or more indicating a substantial level of heterogeneity [32]. To account for differences in baseline HbA1c levels between the studies, we calculated the mean difference between pre- and post-intervention HbA1c levels for the intervention and control groups and the standard deviation (SD) of each difference [33]. Thus, our outcome was the pooled mean difference (MD) in HbA1c levels between the intervention and control groups, along with the 95% confidence interval (CI). We calculated the SD from reported *P* values or CI, as recommended by the Cochrane Collaboration or used the imputation method according to baseline values for missing SDs (we imputed missing SDs according to the pre- intervention values) [32,34].

To assess the potential confounding effect of Heterogeneity, subgroup analyses were performed, according to the characteristics of studies, the HbA1c levels of participants at baseline, the mode of peer support, the frequency of contact, and the length of intervention. Funnel plots were drawn, and Egger tests computed to explore a potential publication bias. A *p* value of less than 0.10 was considered to be statistically significant. A sensitivity analysis was conducted to assess the influence of individual studies on the pooled result, by excluding each study one by one and recalculating the combined MD on the remaining studies.

## Results

### Results of the research and the included studies

**Figure 1:**
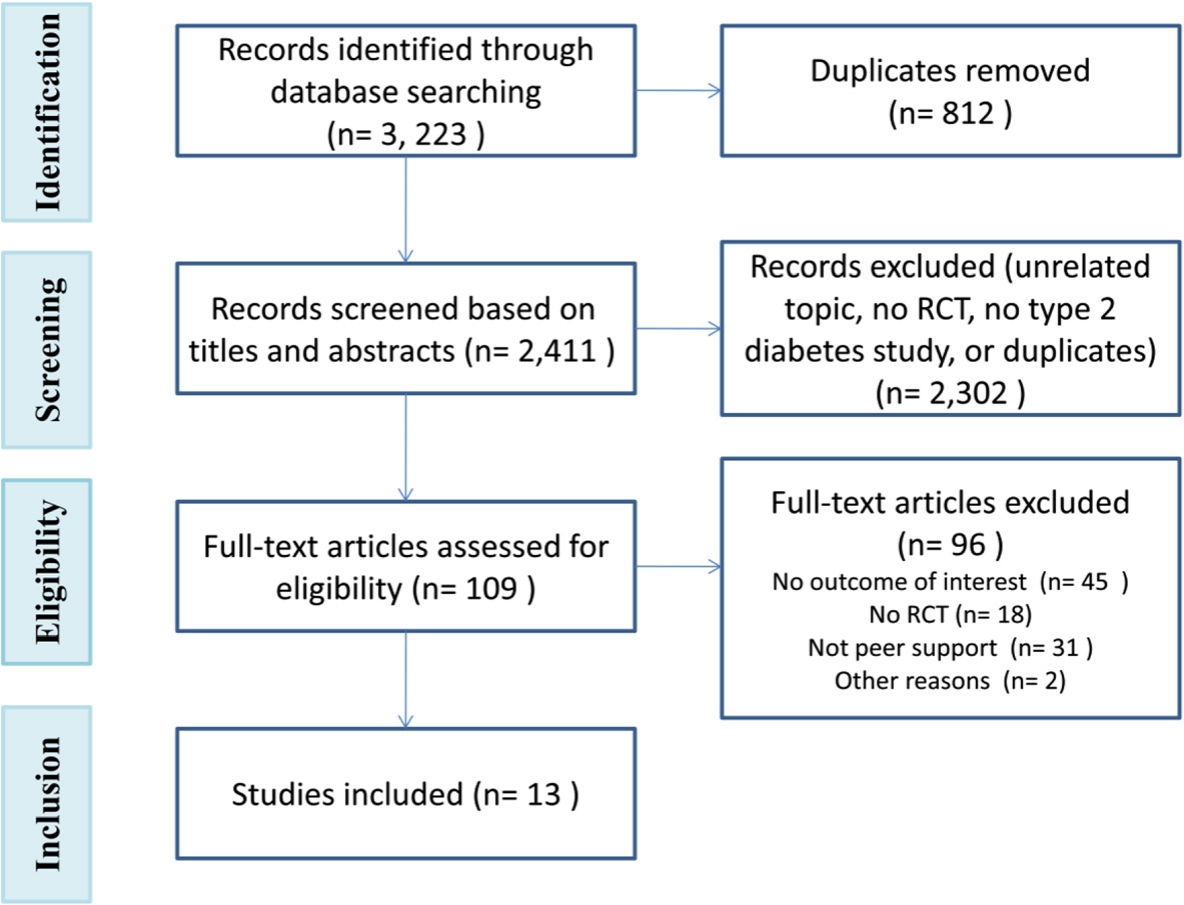
Selection of studies of meta-analysis of peer support interventions.

Initially, 3,223 citations were identified (Figure 1). After initial screening of titles and abstracts, 109 potentially relevant full-text articles were reviewed for eligibility. The review included 13 RCTs [17,18,35-45], involving 2,352 participants.

Details of the included studies, comprising of the characteristics of the study (author, country, study setting) and participants (number, enrollment criteria, age, gender and HbA1c level at baseline), the mode of peer support, the characteristic of intervention of the 13 RCTs are shown in Tables 1 and 2 (at the end of the article). Eleven trials were conducted in the United States, one in Vietnam and one in Ireland. Sample sizes ranged from 38 to 352. The duration of the intervention ranged from 3 to 24 months. The 2,352 participants (63.2% women) were of a mean age of 57.4 years (45.7 - 67.7). The mean HbA1c concentration at baseline was 8.2% (range 6.7 – 10.1).

**Table 1:** **Characteristics of the included studies: study setting, sample size, and study participants**

**First author and year**	**Country**	**Study setting**	**Baseline sample size (intervention/usual care)**	**End of study number (intervention/usual care)**	**Average age (intervention/usual care)**	**Sex (Female) (intervention/usual care)**	**Socioeconomic status**	**Education level**	**Average HbA1c at baseline (%) (intervention/usual care)**
Thom 2013 [35]	USA	Public health clinics	148/151	122/114	54.1/56.3 years	53.0%/51.4%	Low-income	35.6%: < high school education	9.84/10.14
Dang 2013 [36]	Vietnam	Diabetes outpatient clinic	51/51	42/44	NR	NR	NR	NR	7.96/7.85
Prezio 2013 [37]	USA	Community health services clinic	90/90	78/78	47.9/45.7 years	66.7%/54.4%	NR	24.1%: < 6 years; 46%: 6–11 years; 29.9%: ≥12 years.	8.9/8.7
Long 2012 [38]	USA	Philadelphia Veterans Affairs Medical Center	39/39	38/39	60/60 years	0/8%	NR	68%: <12 years	9.8/9.9
Spencer 2011 [39]	USA	2 Communities	84/99	58/69	50/55 year	75%/61%	NR	NR	8.6/8.5
Smith 2011 [17]	Ireland	20 Practices	192/203	166/171	66.1/63.2 years	46%/46%	NR	41%: primary education; 8%: third level education	7.2/7.2
Lorig 2009 [18]	USA	Community	186/159	161/133	67.7/65.4 years	62.4%/66.2%	NR	Mean: 15.9 ± 2.96 years	6.70/6.74
Lorig 2008 [40]	USA	Community	219/198	179/173	52.9/52.8 years	57.1%/67.2%	NR	Mean: 7.68 ± 4.49 years	7.44/7.38
Murrock 2009 [41]	USA	Community	24/22	20/18	58.5/67.1 years	100%/100%	NR	NR	7.7/7.4
Philis-Tsimikas 2011 [42]	USA	Community health centers	104/103	64/81	52.2/49.2 years	66.3%/74.8%	The majority of participants were low-income	54.8%: < 8 years; 45.2%: ≥8 years	10.5/10.3
Lujan 2007 [43]	USA	Community clinic	75/75	71/70	58 years (total)	80% (total)	42%: ≦$10.000 per year	95%: < high school education	8.21/7.71
Samuel-Hodge 2009 [46]	USA	Churches	117/84	102/72	57.0/61.3 years	64%/63%	44%: ≦$10.000 per year	mean: 12.6 ± 0.4 years	7.7/7.9
Feathers 2005 [45]	USA	Community health centers	111/98	91/98	58.5 years/NR	79%/79%	NR	43.0%: < high school; 20.0%: high school; 23.0%: college	8.4/8.4

**Table 2:** **Characteristics of the included studies: characteristics of peer support, frequency, length and theory basis of intervention, and description of usual care group**

**First author and year**	**Mode of peer support**	**Enrollment criteria for Peer coach or CHW**	**Training for peer coach or CHW**	**Group/Individual**	**Frequency of intervention**	**Length of intervention**	**Theory basis**	**Description of usual care group**
Thom 2013 [35]	Peer coach	T2DM who had an HbA1c level of less than 8.5% within the past 6 months	36-hour	Individual (telephone contact and in-person contact)	High	6 months	None	Usual care included all services usually available to patients, including access to a nutritionist and diabetes educator through referral from their primary care clinician.
Dang 2013 [36]	Peer coach	T2DM for one year or more, 30 years old or older, and with HbA1c level in the most recent three months equal to or less than 7%.	Four sessions	Individual (telephone contact)	Moderate	6 months	Social cognitive theory	Follow up at the diabetes outpatient clinic on different dates from the participants in the intervention group to prevent subject contamination.
Prezio 2013 [37]	CHW	Adult female lifelong member of the local Mexican American community, with a high school equivalency (General Educational Development: GED) and certification from the State of Texas as a CHW.	27 h	Individual	Low	12 months	Social cognitive theory	Usual medical care.
Long 2012 [38]	Peer coach or mentors	Diabetes patients whose glucose control had previously been poor but was currently good.	1 hour	Individual (telephone contact)	Low	6 months	Motivational interviewing techniques	Usual care.
Spencer 2011 [39]	CHW	Family health advocates, from the 2 participating communities, where they were ethnically matched with their assigned participants	more than 80 hours	Both	High	6 months	Motivational interviewing and Empowerment theory	Usual care.
Smith 2011 [17]	Peer supporter	T2DM for at least one year; adherent to treatment and behavior change regimens; Capacity and commitment to undergo the training required etc.	Two evening training sessions	Group	Low	24 months	Social support theory	Provided regular recall of patients every three to six months with an annual audit of risk factors.
Lorig 2009 [18]	Peer leaders	Age from 35 to 70 years and came from the same communities as the participants. Most had type 2 diabetes and were not health professionals	4 days	Group	Low	6 months	None	Usual care was representative of care received in urban areas.
Lorig 2008 [40]	Peer leader	Spanish-speaking peer leaders came from the same communities as the participants most had type 2 diabetes and were not health professionals	4 days	Group	Moderate	6 months	None	Usual care.
Murrock 2009 [41]	Peer coach	NR	NR	Group	High	3 months	Social cognitive theory	Usual care group continued with their normal daily routines, medication schedule, diet, and glucose-monitoring regimen.
Philis-Tsimikas 2011 [42]	Peer educators	Individuals with diabetes who exemplified the traits of a natural leader were identified from the patient population and trained as promotoras over a 3-month period	40 h learning, 2 series of classes and then finally taught two series on their own.	Group	High	10 months	None	Usual care.
Lujan 2007 [43]	Peer promotoras	NR	60 hours	Group	High	6 months	Community empowerment	Usual one-on-one patient education by the clinic staff during scheduled medical follow-up visits, which consisted of verbal information and 1 or 2 pamphlets on diabetes self-management skills.
Samuel-Hodge 2009 [46]	Peer counselor	T2DM or having lived with someone diagnosed with diabetes for at least 2 years	over a 1-month period (4 weekly 4-hour sessions)	Both	High	8 months	None	Received standard educational pamphlets by mail.
Feathers 2005 [45]	CHW	African American and Latino community residents	10 weeks	Group	Low	10 months	NR	Usual care.

CHW: community health worker, NR: not reported.

Patients in nine studies attended structured diabetes education sessions conducted by peer supporters weekly or every 2 weeks ( 6 to 8 times, 2 or 2.5 hours every time), covered areas primarily centered on the recommendation by the American Diabetes Association, including the basics of diabetes and its complications, diet, exercise, medication, blood glucose monitoring etc. [17,18,39-43,45,46]. In two of the studies [39,46], patients received individual follow-up by peer supporters to assist them set specific goals and support their progress, after attending all the education sessions. In the other 4 studies [35-38], peer support provided by individual intervention, covered diabetes self-management skills, providing social and emotional support, assisting with lifestyle change and facilitating medication understanding, etc.

### Methodological quality of included studies

A breakdown of study quality is presented in Additional file 1: Table S1. Overall, out of 13 included studies, eight studies adequately described randomization sequence generation, and were free of selective outcome reporting. Ten studies didn’t describe allocation concealment; seven studies didn’t describe blinding to outcome assessment. Most studies were not free of other biases (e.g. lack of strict method to avoid participants in usual care group being contaminated by peer support group). A summary of the risk of bias in included studies is presented in Additional file 2: Figure S1 and Additional file 3: Figure S2.

### Effect of intervention on glycemic control

**Figure 2:**
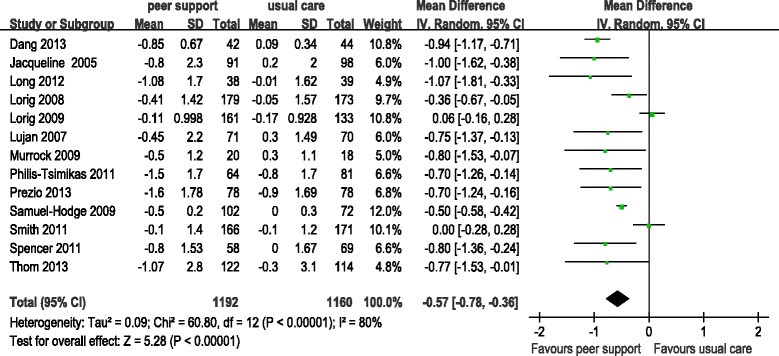
Forest plots show the effect of peer support on the mean difference in HbA1c (%). Mean differences of less than 0 between peer support and usual care groups indicate an effect in favor of peer support.

The impact of the peer support programs on changes in HbA1c level in the intervention and control groups is presented in Figure 2. In the random-effect model, the pooled mean difference in levels between the intervention and control groups was −0.57 (95% CI −0.78 to −0.36), favoring peer support over usual care. No study reported a significant reduction in HbA1c in favor of traditional care. There was significant heterogeneity among the trials regarding changes in HbA1c (*I*^2^ = 80.0%).

### Subgroup analyses

We conducted post hoc subgroup analyses to explore possible differences between studies rendering to their characteristics, the HbA1c levels of participants at baseline, the mode and type of peer support, the frequency of contact, and the length of intervention, etc. (Table 3)

**Table 3:** **Subgroup analyses for the difference in HbA1c with peer support compared with usual care group**

**Study characteristics**	**No. of studies**	**Mean difference of HbA1c, % (95% CI)**	**Heterogeneity**	
			**P**	***I*** ^**2**^ **(%)**
**All studies**	13	−0.57(−0.78 to −0.36)	<0.0001	80.0
**Publication year**				
Before 2010	6	−0.47(−0.76 to −0.17)	<0.0001	82.0
After 2011	7	−0.68-1.05 to −0.31)	<0.0001	79.0
**Study location**				
Medical institution	8	−0.71(−1.05 to −0.37)	<0.0001	77.0
Community or church	5	−0.41(−0.71 to −0.10)	<0.0001	84.0
HbA1c level at baseline*				
≥ 8.5%	5	−0.78(−1.06 to −0.51)	0.94	0.0
7.5% to 8.5%	5	−0.76(−1.05 to −0.47)	0.003	75.0
<7.5%	3	−0.08(−0.32 to 0.16)	0.09	59.0
**Mode of peer support**				
Peer coach	10	−0.51(−0.75 to −0.27)	<0.0001	84.0
Community health worker	3	−0.82(−1.15 to −0.49)	0.92	0.0
**Type of peer support** ^*^				
Individual	4	−0.91(−1.10 to −0.71)	0.81	0.0
Group	7	−0.42(−0.72 to −0.11)	0.0006	74.0
Both	2	−0.52(−0.66 to −0.38)	0.30	8.0
**Frequency of contact**				
High	6	−0.52(−0.60 to −0.44)	0.68	0.0
Moderate	3	−0.75(−1.21 to −0.29)	0.009	79.0
Low	4	−0.32(−0.74 to 0.09)	0.002	80.0
**Length of intervention**				
≦6 months	8	−0.64(−1.01 to −0.27)	<0.0001	85.0
>6 months	5	−0.51(−0.81to 0.20)	0.004	74.0

Note: CI = confidence interval; *p <0.05 (subgroup difference).

The reduction in HbA1c levels were greater among patients with a baseline HbA1c level ≥ 8.5% (MD −0.78 [−1.06 to −0.51]) and between 7.5 ~ 8.5% (MD −0.76 [−1.05 to −0.47]), than patients with a baseline HbA1c level < 7.5% which showed no significant reduction in HbA1c levels compared with usual care (MD −0.08 [−0.32 to 0.16]). (p < 0.05 for subgroup difference, Additional file 4: Figure S3).

Patients in four studies were provided with individual intervention and responded by a greater reduction in HbA1c level (MD −0.91 [−1.10 to −0.71]) in comparison with patients provided with group session education (MD −0.42 [−0.72 to −0.11] ) or a combination of group and individual education (MD −0.52 [−0.66 to −0.38]). (P < 0.05 for subgroup difference, Additional file 5: Figure S4).

Programs with a high or moderate frequency of contact reported a significant reduction in HbA1C levels compared with usual care (MD −0.52 [−0.60 to −0.44]) and −0.75 [−1.21 to −0.29]). Nevertheless, programs with a low frequency of contact showed no significant reduction in HbA1c level compared with usual care (MD −0.32 [−0.74 to 0.09]; Additional file 6: Figure S5).

We found no major differences in HbA1c levels relative to publication year, the mode of peer support, study location or the duration of intervention (Table 3).

### Publication bias and sensitivity analyses

We explored the possibility of publication bias for the included 13 studies. The funnel plot for the outcome HbA1c showed a slight asymmetry, using the Egger test (*P* = 0.014), indicating a potential publication bias (Additional file 7: Figure S6).

In order to investigate the reliability of the results, we analyzed their sensitivity. After excluding each of the studies one at a time, the analyses did not detect any influence of one single study on the overall results (Additional file 8: Figure S7).

## Discussions

Our meta-analysis suggested that peer support has a favorable effect on improving glycemic control, with a pooled mean reduction of 0.57% in HbA1c levels compared with usual care. This study has important implications for current clinical and public health practice and research. Glycaemic control is an important predictor of many of the chronic complications of diabetes [47,48]. According to the UK Prospective Diabetes Study, each 1% reduction in HbA1c over 10 years is associated with reductions in risk of up to 21% for any end point related to diabetes, 21% for deaths related to diabetes, 14% for myocardial infarctions and 37% for micro-vascular complications [48]. Thus, the improvement in HbA1c of 0.57% is clinically significant. Moreover, this finding are probably underestimated because the usual care provided in control groups in RCTs is often better than that provided in clinical practice. Some studies included in our meta-analysis permitted patients in the control group to contact the medical team or be contacted by them during follow-up in addition to usual care [17,35,43].

Our findings showed that peer support is more effective for patients who have poor glycemic control (mean HbA1c ≥ 7.5% at baseline) than for those with better glycemic control. These results might be partly because patients with lower HbA1c levels at baseline had already reached a floor effect, leaving little room for improvement after intervention. Thus, peer support could be particularly effective if targeted at patients with non-stabilized diabetes. It is worth mentioning that such patients have a higher risk of developing complications and so would probably derive greater long-term benefits from peer support.

Of the 13 included RCTs, most were peer-led group self-management education rather than individual intervention. To date, many peer-led group intervention programs followed a model that was first developed and evaluated by Kate Lorig et al. (http://patienteducation.stanford.edu): the Chronic Disease Self-Management Program (CDSMP). The CDSMP is a program for patients with different chronic conditions including diabetes given in 2.5-hour sessions once a week over 6 weeks. Peer supporters offer the courses in an interactive manner designed to enhance participants’ confidence in their ability to execute specific self-care tasks. Compared with traditional CDSMP led by professionals, peer-led interventions are more easily held outside of normal working hours, allowing more courses to be offered at varying times. The results indicated the effectiveness of peer-led group self-management education, with a significant decrease of mean HbA1c value of – 0.42%. However, some participants face challenges in attending structured face-to-face meetings, and it was also difficult to summon all the participants together at the same time.

In addition to group intervention programs, there were some individual peer-led interventions, which seem more informal and flexible than structured group interventions. By individual intervention, peer supporters meet other patients and listen, discuss concerns and provide support to them. These peer supporters usually receive initial training of 8 to 32 hours with the training focusing on communication skills, including empathic listening, helping participants clarify their values and life goals, problem-solving and assertiveness. The results of our meta-analysis showed that the individual intervention might be more effective than structured group intervention and group education followed by individual on-going support. Moreover, three of the four peer-led individual intervention RCTs were conducted by telephone, which is helpful in avoiding distance barriers and allows for frequent contact with patients at a lower cost. Therefore, if carefully designed and implemented, telephone-based peer support might be a satisfactory choice for diabetes management and should be introduced on a large scale.

In order to explore the effect of program intensity on its effectiveness, we explored the length of intervention and the frequency of patients’ contact. We didn’t find any significant variance linked to the length of intervention; however, the frequency of contact seemed to be a key feature of the effectiveness. Peer support with low frequency of contact showed no significant change on its effectiveness, whereas moderate and high frequency of contact pointed to a significant improvement of glycemic control. This result means that only peer-support with moderate or intensive intervention should be implemented. We also explored the mode of peer support on the programme’s effectiveness but didn’t find any significant difference between the impact of community health workers or peer coaches. Thus, both of them could be potential peer supporters based on the different settings and populations.

The strengths of the study included a comprehensive, systematic review of the literature; we used a broad search strategy to capture all relevant information. Furthermore, we included only RCTs and several recent studies. Therefore, our estimate is probably more precise than that in previous studies.

Our study, nonetheless, has some limitations. Firstly, a high level of statistical heterogeneity was noted in our review. Therefore, we conducted subgroup analyses to explore it and used random-effect model that enabled heterogeneity to be accounted for in the analysis. However, it is hard to conduct sub-group analysis for the socioeconomic status and the educational level of the participants due to the insufficient information, which indicate that information on the socioeconomic status and education level of participants should be reported in future trials. Secondly, as in all meta-analysis, the possibility of publication bias and selection bias is of concern. Thirdly, the number of studies on CHWs is limited, further epidemiological studies are needed to be done to accumulate more evidence. Finally, most of the studies met our inclusion criteria conducted in the United States, which might influence the generalizability of the findings to other types of geographic areas.

## Conclusions

In conclusion, peer support models provide a potentially flexible means for diabetes self-management education or on-going support programs. Practical components need a moderate or high frequency of patient contact with targeted patients those of poor glycemic control. Priority should be given to programs with moderate or high contact of target patients, with poor glycemic control, rather than programs with low frequency of patients’ contact that target the overall population of patients with T2DM. In addition, more telephone-based peer support programs are needed to explore the long-term efficacy on glycemic control.

Considering most of the included studies were implemented in USA and were at a potential risk of bias, as many studies were rated as ‘unclear’ because of a lack of information, especially on allocation concealment, blinding to outcomes data and other biases (e.g. strict methods to avoid participants in usual care group being contaminated by peer support), all of which might reduce the internal validity of the studies. Therefore, well-designed high-quality trials are needed to demonstrate the efficacy of peer support in different settings, especially in low-income countries.

## Additional files

Additional file 1: Table S1. Risk of bias of included studies (ordered by study ID). Additional file 2: Figure S1. Risk of the bias summary: review authors’ judgments about each risk of bias item for each included study. Additional file 3: Figure S2. Risk of bias graph: review authors’ judgments about each risk of bias item presented as percentages across all included studies. Additional file 4: Figure S3. Forest plots show the effect of peer support on the mean difference in HbA1c (%) by different HbA1c level at baseline. Additional file 5: Figure S4. Forest plots show the effect of peer support on the mean difference in HbA1c (%) by different type of peer support. Additional file 6: Figure S5. Forest plots show the effect of peer support on the mean difference in HbA1c (%) by different frequency of peer support. Additional file 7: Figure S6. Funnel plot of meta-analysis of the effect of peer support on the mean difference in HbA1c level among patients with type 2 diabetes. Additional file 8: Figure S7. Sensitivity analysis (leave-one-out) of meta-analysis of the effect of peer support on the mean difference in HbA1c level among patients with type 2 diabetes.
