# Supplementary material for: Effectiveness of peer support for improving glycaemic control in patients with type 2 diabetes: a meta-analysis of randomized controlled trials
Source: BMC Public Health. 2015 May 6;15:471. doi: 10.1186/s12889-015-1798-y (PMC4425885; doi:10.1186/s12889-015-1798-y)
Supplement: Additional file 1: Table S1. — Risk of bias of included studies (ordered by study ID). [file 12889_2015_1798_MOESM1_ESM.doc]

**Table S1.Risk of bias of included studies (ordered by study ID)**

| **Risk of bias- Thom 2013** |  |  |
| --- | --- | --- |
| Item | Support for judgment | Authors’ judgment |
| Adequate sequence generation? | Yes | Low risk |
|  | Quote: “Patients were assigned to the usual care or peer-coaching study arm using randomly ordered opaque envelopes.” |  |
| Allocation concealment? | Unclear | Unclear risk |
|  | Comment: no information. |  |
| Blinding of outcome assessment? | Unclear | Unclear risk |
|  | Comment: no information. |  |
| Incomplete outcome data addressed? | Yes | Low risk |
|  | Quote: “Patients who dropped out were on average younger, more likely to smoke, and less likely to report having hyperlipidemia, but did not otherwise differ significantly from patients remaining in the study.” |  |
| Free of selective reporting? | Yes | Low risk |
|  | Comment: The main outcome (HbA1c) was stated. |  |
| Free of other bias? | Unclear | Unclear risk |
|  | Quote: “Because patients receiving peer coaching and those receiving usual care were seen at the same clinics, and often by the same clinicians, it is possible that the presence of peer coaching influenced (contaminated) the usual care group. Such an effect would be expected to make the groups more alike and make it more difficult to show a difference between groups.” |  |

| **Risk of bias - Dang 2013** |  |  |
| --- | --- | --- |
| Item | Support for judgment | Authors’ judgment |
| Adequate sequence generation? | Yes | Low risk |
|  | Quote:“Participants were randomly assigned to either the control or intervention group by flipping a coin.” |  |
| Allocation concealment? | Unclear | Unclear risk |
|  | Comment: no information. |  |
| Blinding of outcome assessment? | Unclear | Low risk |
|  | Comment: no information. |  |
| Incomplete outcome data addressed? | Unclear | Unclear risk |
|  | Comment: no information on reasons for missing data provided. |  |
| Free of selective reporting? | Yes | Low risk |
|  | Comment: The main outcome (HbA1c) was stated. |  |
| Free of other bias? | Yes | Low risk |
|  | Quote:“The participants in the control group were appointed to follow up at the diabetes outpatient clinic on different dates from the participants in the intervention group to prevent subject contamination. ” |  |

| **Risk of bias - Prezio 2013** |  |  |
| --- | --- | --- |
| Item | Support for judgment | Authors’ judgment |
| Adequate sequence generation? | Yes | Low risk |
|  | Quote:“Participants were randomly assigned to either the control or intervention group by using a computer generated randomization schedule.” |  |
| Allocation concealment? | Unclear | Unclear risk |
|  | Comment: no information. |  |
| Blinding of outcome assessment? | Yes | Low risk |
|  | Quote:“HbA1c was measured using a Bayer DCA 2000 + Analyzer, rigorously maintained for quality control. Blood pressure was.” |  |
| Incomplete outcome data addressed? | Yes | Low risk |
|  | Comment: reasons for missing data provided in Fig.1 and unlikely to be related to true outcome. |  |
| Free of selective reporting? | Yes | Low risk |
|  | Comment: The main outcome (HbA1c) was stated. |  |
| Free of other bias? | Unclear | Unclear risk |
|  | Comment: it is possible that the presence of peer coaching influenced the usual care group. |  |

| **Risk of bias - Long 2012** |  |  |
| --- | --- | --- |
| Item | Support for judgment | Authors’ judgment |
| Adequate sequence generation? | Yes | Low risk |
|  | Comment: The authors using the random-number generator function of Microsoft Excel 2007. |  |
| Allocation concealment? | Yes | Low risk |
|  | Quote:“The envelopes were sealed, shuffled, and stacked, and the research assistant took the top envelope after consent was obtained to determine group assignment. Neither blocking nor stratiﬁcation was used in the process.” |  |
| Blinding of outcome assessment? | Yes | Low risk |
|  | Comment: the HbA1c was measured objectively |  |
| Incomplete outcome data addressed? | Yes | Low risk |
|  | Quote: “The multiple imputation method assumes that data are missing at random or that depends on observed variables only.” |  |
| Free of selective reporting? | Yes | Low risk |
|  | Comment: The main outcome (HbA1c) was stated. |  |
| Free of other bias? | Unclear | Unclear risk |
|  | Comment: it is possible that the presence of peer coaching influenced the usual care group. |  |

| **Risk of bias - Spencer 2011** |  |  |
| --- | --- | --- |
| Item | Support for judgment | Authors’ judgment |
| Adequate sequence generation? | Unclear | Unclear risk |
|  | Comment: there was no information provided about the adequacy of Random sequence generation. |  |
| Allocation concealment? | Unclear | Unclear risk |
|  | Comment: no information. |  |
| Blinding of outcome assessment? | Yes | Low risk |
|  | Comment: the data analysts were blinded. |  |
| Incomplete outcome data addressed? | Yes | Low risk |
|  | Quote: “Because we were missing data for some participants’ HbA1c values, we conducted a second analysis that imputed missing data.” |  |
| Free of selective reporting? | Yes | Low risk |
|  | Comment: The main outcome (HbA1c) was stated. |  |
| Free of other bias? | No | High risk |
|  | Quote: “the communitywide healthy eating and physical activity programs available to and promoted among both groups as part of REACH Detroit’s community-level programs……”, thus, it is possible that the presence of peer coaching influenced the usual care group. |  |

| **Risk of bias - Smith 2011** |  |  |
| --- | --- | --- |
| Item | Support for judgment | Authors’ judgment |
| Adequate sequence generation? | No | High risk |
|  | Quote:“Practices were stratified by practice size and the presence of existing structured diabetes care and were then allocated to control or intervention group by an independent statistician using minimization.”, thus, this study was a cluster randomized controlled trial. |  |
| Allocation concealment? | Unclear | Unclear risk |
|  | Comment: no information. |  |
| Blinding of outcome assessment? | Unclear | Unclear risk |
|  | Comment: no information. |  |
| Incomplete outcome data addressed? | Unclear | Unclear risk |
|  | Comment: no information on reasons for missing data provided. |  |
| Free of selective reporting? | Yes | Low risk |
|  | Comment: primary outcome measure was stated. |  |
| Free of other bias? | Unclear | Unclear risk |
|  | Comment: it is possible that the presence of peer coaching influenced the usual care group. |  |

| **Risk of bias - Lorig 2009** |  |  |
| --- | --- | --- |
| Item | Support for judgment | Authors’ judgment |
| Adequate sequence generation? | Yes | Low risk |
|  | Quote: “After participants had applied to attend at a specific site and completed baseline questionnaires, randomization was performed using random number tables.” |  |
| Allocation concealment? | Unclear | Unclear risk |
|  | Comment: no information. |  |
| Blinding of outcome assessment? | Unclear | Unclear risk |
|  | Comment: no information. |  |
| Incomplete outcome data addressed? | Yes | Low risk |
|  | Quote: “All analyses were done using both actual data collected and intent-to-treat methodology—based on substituting last acquired data for missing data…...” |  |
| Free of selective reporting? | Yes | Low risk |
|  | Comment: The main outcome (HbA1c) was stated. |  |
| Free of other bias? | No | High risk |
|  | Quote: “Because there were few differences in the baseline values of outcome variables between the 6-month intervention no completers and usual-care control group no completers ...Thus, it is not very likely that no completion was an important factor affecting the 6-month randomized study.” |  |

| **Risk of bias- Lorig 2008** |  |  |
| --- | --- | --- |
| Item | Support for judgment | Authors’ judgment |
| Adequate sequence generation? | Unclear | Unclear risk |
|  | Quote: “Following baseline data collection, most study participants were randomized to three groups.”, however, no detail information was described on randomization. |  |
| Allocation concealment? | Unclear | Unclear risk |
|  | Comment: no information. |  |
| Blinding of outcome assessment? | Unclear | Unclear risk |
|  | Comment: no information. |  |
| Incomplete outcome data addressed? | Unclear | Unclear risk |
|  | Comment: no information on reasons for missing data provided. |  |
| Free of selective reporting? | Yes | Low risk |
|  | Comment: The main outcome (HbA1c) was stated. |  |
| Free of other bias? | Unclear | Unclear risk |
|  | Quote: “There was a significant difference in the percentage of female participants between the control and intervention groups (67.2 vs. 57.1%, P 0.034; Table 1)....”, and it is possible that the presence of peer coaching influenced the usual care group. |  |

| **Risk of bias - Murrock 2009** |  |  |
| --- | --- | --- |
| Item | Support for judgment | Authors’ judgment |
| Adequate sequence generation? | Yes | Low risk |
|  | Quote: “Based on a coin flip, 36 women were randomized to the usual care group and 34 women were randomized to the dance group.” |  |
| Allocation concealment? | Unclear | Unclear risk |
|  | Comment: no information. |  |
| Blinding of outcome assessment? | Unclear | Unclear risk |
|  | Comment: no information. |  |
| Incomplete outcome data addressed? | Unclear | Unclear risk |
|  | Comment: no information on reasons for missing data provided. |  |
| Free of selective reporting? | Yes | Low risk |
|  | Comment: The main outcome (HbA1c) was stated. |  |
| Free of other bias? | Unclear | Unclear risk |
|  | Comment: it is possible that the presence of peer coaching influenced the usual care group. |  |

| **Risk of bias - Philis-Tsimikas 2011** |  |  |
| --- | --- | --- |
| Item | Support for judgment | Authors’ judgment |
| Adequate sequence generation? | Yes | Low risk |
|  | Quote: “Blocked random assignment with equal allocation was used to assign participants to the control or Project Dulce groups using a randomly generated numbers sequence.” |  |
| Allocation concealment? | No | High risk |
|  | Comment: Participants were informed of their group allocation after the baseline assessment. |  |
| Blinding of outcome assessment? | Unclear | Unclear risk |
|  | Comment: no information. |  |
| Incomplete outcome data addressed? | Unclear | Unclear risk |
|  | Comment: no information on reasons for missing data provided. |  |
| Free of selective reporting? | Yes | Low risk |
|  | Comment: The main outcome (HbA1c) was stated. |  |
| Free of other bias? | Unclear | Unclear risk |
|  | Comment: it is possible that the presence of peer coaching influenced the usual care group. |  |

| **Risk of bias - Lujan 2007** |  |  |
| --- | --- | --- |
| Item | Support for judgment | Authors’ judgment |
| Adequate sequence generation? | Unclear | Unclear risk |
|  | Quote: “The 150 consenting participants who were selected were randomized into an intervention and a usual-care control group.” , but without detail information. |  |
| Allocation concealment? | Yes | Low risk |
|  | Quote: “A trained bilingual research assistant, masked to the intervention and group assignment.” |  |
| Blinding of outcome assessment? | Unclear | Unclear risk |
|  | Comment: Quote: “A finger-stick procedure was used to obtain the blood, and a Bayer 2000 analyzer was used to analyze the sample.”, however, it is unclear whether the data analyst was blinded. |  |
| Incomplete outcome data addressed? | Yes | Low risk |
|  | Comment: reasons for missing data provided and unlikely to be related to true outcome. |  |
| Free of selective reporting? | Yes | Low risk |
|  | Comment: The main outcome (HbA1c) was stated. |  |
| Free of other bias? | Unclear | Unclear risk |
|  | Comment: it is possible that the presence of peer promoter influenced the usual care group. |  |

| **Risk of bias - Samuel-Hodge 2009** |  |  |
| --- | --- | --- |
| Item | Support for judgment | Authors’ judgment |
| Adequate sequence generation? | Yes | Low risk |
|  | Quote: “A research assistant accomplished randomization by opening the next envelope from a set of sequentially numbered sealed envelopes containing study group assignment as determined by random numbers generated by a statistical consultant using a personal computer.” |  |
| Allocation concealment? | Unclear | Unclear risk |
|  | Comment: no information. |  |
| Blinding of outcome assessment? | Yes | Low risk |
|  | Quote: “Laboratory staff assessing A1C were masked to the participants’ study group. ” |  |
| Incomplete outcome data addressed? | Yes | Low risk |
|  | Comment: reasons for missing data provided and unlikely to be related to true outcome. |  |
| Free of selective reporting? | Yes | Low risk |
|  | Comment: The main outcome (HbA1c) was stated. |  |
| Free of other bias? | Unclear | Unclear risk |
|  | Quote:“ Except for a difference in age, with SI participants being about 3 years younger, study participants were similar as assessed on baseline variables.”, and it is possible that the presence of peer coaching influenced the usual care group. |  |

| **Risk of bias - Feathers 2005** |  |  |
| --- | --- | --- |
| Item | Support for judgment | Authors’ judgment |
| Adequate sequence generation? | No | High risk |
|  | Comment: HbA1c of patients in control group were abstracted from the medical charts of a random sample of insured non- REACH Detroit African American and Latino patients with type 2 diabetes. |  |
| Allocation concealment? | Unclear | Unclear risk |
|  | Comment: no information. |  |
| Blinding of outcome assessment? | No | High risk |
|  | Comment: the HbA1c values of participants in control group were abstracted from medical charts. |  |
| Incomplete outcome data addressed? | Unclear | Unclear risk |
|  | Comment: no reasons for missing data provided. |  |
| Free of selective reporting? | Yes | Low risk |
|  | Comment: The main outcome (HbA1c) was stated. |  |
| Free of other bias? | No | High risk |
|  | Quote: “It is more difficult to draw conclusions about causality from no experimental designs that may be subject to selection bias.” |  |
