# Supplementary material for: Effectiveness of peer support for improving glycaemic control in patients with type 2 diabetes: a meta-analysis of randomized controlled trials
Source: BMC Public Health. 2015 May 6;15:471. doi: 10.1186/s12889-015-1798-y (PMC4425885; doi:10.1186/s12889-015-1798-y)
Supplement: Additional file 2: Figure S1. — Risk of the bias summary: review authors’ judgments about each risk of bias item for each included study. [file 12889_2015_1798_MOESM2_ESM.doc]

Figure S1. Risk of bias summary: review authors’ judgments about each risk of bias item for each included study.
