# Supplementary material for: Effectiveness of peer support for improving glycaemic control in patients with type 2 diabetes: a meta-analysis of randomized controlled trials
Source: BMC Public Health. 2015 May 6;15:471. doi: 10.1186/s12889-015-1798-y (PMC4425885; doi:10.1186/s12889-015-1798-y)
Supplement: Additional file 3: Figure S2. — Risk of bias graph: review authors’ judgments about each risk of bias item presented as percentages across all included studies. [file 12889_2015_1798_MOESM3_ESM.doc]

Figure S2. Risk of bias graph: review authors’ judgments about each risk of bias item presented as percentages across all included studies.
