# Supplementary material for: Effectiveness of peer support for improving glycaemic control in patients with type 2 diabetes: a meta-analysis of randomized controlled trials
Source: BMC Public Health. 2015 May 6;15:471. doi: 10.1186/s12889-015-1798-y (PMC4425885; doi:10.1186/s12889-015-1798-y)
Supplement: Additional file 5: Figure S4. — Forest plots show the effect of peer support on the mean difference in HbA1c (%) by different type of peer support. [file 12889_2015_1798_MOESM5_ESM.doc]

Figure S4.Forest plots show the effect of peer support on the mean difference in HbA1c (%) by different type of peer support. Mean differences of less than 0 between peer support and usual care groups indicate an effect in favor of peer support.
