# Supplementary material for: Effectiveness of peer support for improving glycaemic control in patients with type 2 diabetes: a meta-analysis of randomized controlled trials
Source: BMC Public Health. 2015 May 6;15:471. doi: 10.1186/s12889-015-1798-y (PMC4425885; doi:10.1186/s12889-015-1798-y)
Supplement: Additional file 7: Figure S6. — Funnel plot of meta-analysis of the effect of peer support on the mean difference in HbA1c level among patients with type 2 diabetes. [file 12889_2015_1798_MOESM7_ESM.doc]

Figure S6.Funnel plot of meta-analysis of the effect of peer support on the mean difference in HbA1c value among patients with type 2 diabetes.
